# Supplementary material for: Aspergillus in Critically Ill COVID-19 Patients: A Scoping Review
Source: J Clin Med. 2021 Jun 2;10(11):2469. doi: 10.3390/jcm10112469 (PMC8215643; doi:10.3390/jcm10112469)
Supplement: Supplementary file 1 [file jcm-10-02469-s001.zip › jcm-1212371-supplementary.pdf]

Supplementary Files and Table

Supplementary File S1

Search terms used regarding the association between Aspergillus findings and COVID-19:

(Coronavirus OR COVID OR SARS OR Sars-CoV-2 OR Covid-19) AND (‘’aspergillosis’’[MeSH] OR aspergillus OR galactomannan OR galactomannan index).

Supplementary File S2.

Newcastle-Ottawa Quality Assessment Form for Cohort Studies adapted for this study
Note: A study can be given a maximum of one star for each numbered item within the Selection and Outcome categories. A maximum of two stars can be given for Comparability.

Selection
1) Representativeness of the exposed cohort (i.e. clear description on why Aspergillus test was performed)

a) Representative ***(one star)***

b) Selected group

c) No description of the derivation of the cohort

2) Selection of the non-exposed cohort

a) Drawn from the same community as the exposed cohort ***(one star)***

b) Drawn from a different source

c) No description of the derivation of the non-exposed cohort

3) Ascertainment of exposure

a) The reason why samples were obtained was described ***(one star)***

b) How samples were obtained and critical comments on the representativeness of samples were described ***(one star)***

b) No description

Comparability
1) Comparability of cohorts on the basis of the design or analysis controlled for confounders

a) Cohorts are comparable on the basis of the design or analysis controlled for confounders The study controls for age, sex, or other factors ***(one star)***

Outcome
1) Assessment of outcome (mortality)

a) Performed in exposed (i.e. patients with aspergillosis) and in non-exposed ***(one star)***

2) Adequacy of follow-up of cohorts

a) Complete follow up- all subject accounted for, i.e. end date of the cohort follow-up was clearly defined ***(one star)***

Thresholds for converting the Newcastle-Ottawa scales to AHRQ standards (good, fair, and poor):
Good quality: 3 or 4 stars in selection domain AND 1 star in comparability domain AND 2 stars in outcome/exposure domain
Fair quality: 2 stars in selection domain AND 1 star in comparability domain AND 1 or 2 stars in outcome/exposure domain
Poor quality: 0 or 1 star in selection domain OR 0 stars in comparability domain OR 0 or 1 stars in outcome/exposure domain

Supplementary Table S1. Quality assessment results of included studies

First author (ref)	Selection			Comparability	Outcome		Conclusion	Remarks
	Representativeness exposed	Selection of the non-exposed cohort	Ascertainment of exposure	Comparability	Mortality rate was assessed in exposure and non-exposure	Adequacy follow-up		
Bartoletti (1)	*	*	**	*	-	*	Fair	No analysis of mortality in ICU patients without systematic respiratory sample
Dellière (2)	*	*	-	-	-	-	Poor	Only patients with respiratory samples available were included
Fekkar (3)	*	*	**	*	*	*	Good	
Gangneux (4)	*	*	*	*	*	*	Good	All types of respiratory samples were included, but no galactomannan was performed
Gonzalo Segrelles-Calvo (5)	*	-	*	*	*	-	Fair	It was mentioned 'Patients without a microbiological diagnosis of SARS-CoV-2 or IFI, and those from whom the planned data was not collected were excluded from the database.'<br>A. flavus was also included in the analysis
Lewis White (6)	-	-	*	-	-	-	Poor	No clear definition on how the study samples were selected.

Razazi (7)	*	*	**	*	*	*	Good	Study aim was to compare risk factors for development of ventilator associated pneumonia vin COVID-19 vs. non COVID-19 patients
Versyck (8)	*	*	**	-	-	-	Poor	Limited information in non-cases.

References to the Supplementary Tables (Other Reference Order was Used in the Paper)

1. Bartoletti M, Pascale R, Cricca M, Rinaldi M, Maccaro A, Bussini L, et al. Epidemiology of Invasive Pulmonary Aspergillosis Among Intubated Patients With COVID-19: A Prospective Study. Clin Infect Dis. 2020;

2. Dellière S, Dudoignon E, Fodil S, Voicu S, Collet M, Oillic PA, et al. Risk factors associated with COVID-19-associated pulmonary aspergillosis in ICU patients: a French multicentric retrospective cohort. Clin Microbiol Infect. 2021;

3. Fekkar A, Lampros A, Mayaux J, Poignon C, Demeret S, Constantin J-M, et al. Occurrence of Invasive Pulmonary Fungal Infections in Patients with Severe COVID-19 Admitted to the ICU. Am J Respir Crit Care Med [Internet]. 2021;203:307–17.

4. Gangneux JP, Reizine F, Guegan H, Pinceaux K, Le Balch P, Prat E, et al. Is the covid-19 pandemic a good time to include aspergillus molecular detection to categorize aspergillosis in icu patients? A monocentric experience. J Fungi. 2020;

5. Segrelles-Calvo G, Araújo GRS, Llopis-Pastor E, Carrillo J, Hernández-Hernández M, Rey L, et al. Prevalence of opportunistic invasive aspergillosis in COVID-19 patients with severe pneumonia. Mycoses. 2021;

6. White PL, Dhillon R, Cordey A, Hughes H, Faggian F, Soni S, et al. A National Strategy to Diagnose Coronavirus Disease 2019–Associated Invasive Fungal Disease in the Intensive Care Unit. Clin Infect Dis. 2020;

7. Razazi K, Arrestier R, Haudebourg AF, Benelli B, Carteaux G, Decousser J -W, et al. Risks of ventilator-associated pneumonia and invasive pulmonary aspergillosis in patients with viral acute respiratory distress syndrome related or not to Coronavirus 19 disease. Crit Care. 2020;

8. Versyck M, Zarrougui W, Lambiotte F, Elbeki N, Saint-Leger P. Invasive pulmonary aspergillosis in COVID-19 critically ill patients: Results of a French monocentric cohort. J Mycol Med [Internet]. 2021 Feb 16 [cited 2021 Mar 18];31(2):101122.
